# The Fire and Tree Mortality Database, for empirical modeling of individual tree mortality after fire

**DOI:** 10.1038/s41597-020-0522-7

**Published:** 2020-06-22

**Authors:** C. Alina Cansler, Sharon M. Hood, J. Morgan Varner, Phillip J. van Mantgem, Michelle C. Agne, Robert A. Andrus, Matthew P. Ayres, Bruce D. Ayres, Jonathan D. Bakker, Michael A. Battaglia, Barbara J. Bentz, Carolyn R. Breece, James K. Brown, Daniel R. Cluck, Tom W. Coleman, R. Gregory Corace, W. Wallace Covington, Douglas S. Cram, James B. Cronan, Joseph E. Crouse, Adrian J. Das, Ryan S. Davis, Darci M. Dickinson, Stephen A. Fitzgerald, Peter Z. Fulé, Lisa M. Ganio, Lindsay M. Grayson, Charles B. Halpern, Jim L. Hanula, Brian J. Harvey, J. Kevin Hiers, David W. Huffman, MaryBeth Keifer, Tara L. Keyser, Leda N. Kobziar, Thomas E. Kolb, Crystal A. Kolden, Karen E. Kopper, Jason R. Kreitler, Jesse K. Kreye, Andrew M. Latimer, Andrew P. Lerch, Maria J. Lombardero, Virginia L. McDaniel, Charles W. McHugh, Joel D. McMillin, Jason J. Moghaddas, Joseph J. O’Brien, Daniel D. B. Perrakis, David W. Peterson, Susan J. Prichard, Robert A. Progar, Kenneth F. Raffa, Elizabeth D. Reinhardt, Joseph C. Restaino, John P. Roccaforte, Brendan M. Rogers, Kevin C. Ryan, Hugh D. Safford, Alyson E. Santoro, Timothy M. Shearman, Alice M. Shumate, Carolyn H. Sieg, Sheri L. Smith, Rebecca J. Smith, Nathan L. Stephenson, Mary Stuever, Jens T. Stevens, Michael T. Stoddard, Walter G. Thies, Nicole M. Vaillant, Shelby A. Weiss, Douglas J. Westlind, Travis J. Woolley, Micah C. Wright

**Affiliations:** 1 School of Environmental and Forest Sciences, University of Washington, Box 352100, Seattle, WA 98195 USA; 2 USDA Forest Service, Rocky Mountain Research Station, Fire, Fuel, and Smoke Science Program, 5775 US Highway 10 W, Missoula, MT 59808 USA; 3 Tall Timbers Research Station, 13093 Henry Beadel Drive, Tallahassee, FL 32312 USA; 4 U.S. Geological Survey, Western Ecological Research Center, 1655 Heindon Road, Arcata, CA 95521 USA; 5 Department of Geography, University of Colorado Boulder, Guggenheim 110, 260 UCB, Boulder, Colorado, 80309-0260 USA; 6 Biological Sciences, Dartmouth College, 78 College St., Hanover, NH 03755 USA; 7 Woodsrun Forest Products, 310 W 3rd Ave, Colfax Wi, 54730 USA; 8 USDA Forest Service, Rocky Mountain Research Station, 240 West Prospect Rd, Fort Collins, CO 80526 USA; 9 USDA Forest Service, Rocky Mountain Research Station, 860 N 1200 E, Logan, UT 84321 USA; 10 Department of Horticulture, Oregon State University, 4017 ALS Bldg., Corvallis, OR 97331 USA; 11 USDA Forest Service, Forest Health Protection, 2550 Riverside Drive, Susanville, CA 96130 USA; 12 USDA Forest Service, Forest Health Protection, 200 W. T. Weaver Blvd, Asheville, NC 28804 USA; 13 Forestry Assistance Program, State of Michigan, 1900 M-32 West, Alpena, Michigan 49707 USA; 14 School of Forestry, Northern Arizona University, 200 E Pine Knoll Dr., Flagstaff, AZ 86011 USA; 15 New Mexico State University, MSC 3AE, P.O. Box 30003, Las Cruces, NM 88003 USA; 16 USDA Forest Service, PNW Research Station, 400 N 34th St., Ste 201, Seattle, WA 98103 USA; 17 Ecological Restoration Institute, Northern Arizona University, PO Box 15017, Flagstaff, AZ 86011-5017 USA; 18 U.S. Geological Survey, Western Ecological Research Center, 47050 Generals Highway, Three Rivers, CA 93271 USA; 19 Department of Biology, Utah State University, 5305 Old Main Hill, Logan, UT 84322 USA; 20 USDA Forest Service, Forest Health Protection 1133 N. Western Ave., Wenatchee, WA 98801 USA; 21 OSU Extension Silviculture & Fire Specialist, 8692 Peavy Arboretum Rd., Corvallis, OR 97330 USA; 22 Department of Statistics, 239 Weniger Hall, Oregon State University, Corvallis, OR 97333 USA; 23 Bureau of Land Management, 777 NW Garden Valley Blvd., Roseburg, OR 97471 USA; 24 USDA Forest Service, Southern Research Station, 320 Green Street, Athens, GA 30602 USA; 25 National Park Service, Fire Management Program Center, 3833 S. Development Ave, Boise, ID 83705 USA; 26 USDA Forest Service, Southern Research Station, Upland Hardwood Ecology and Management, 1577 Brevard Rd., Asheville, NC 28806 USA; 27 University of Idaho, Natural Resources and Society, 875 Perimeter Drive, Moscow, Idaho 83844 USA; 28 University of California, Merced, 5200 N Lake Rd, Merced, CA 95343 USA; 29 North Cascades National Park Service Complex, 810 State Route 20, Sedro-Woolley, WA 98284 USA; 30 US Geological Survey, Western Geographic Science Center, Boise, ID 83706 USA; 31 Department of Ecosystem Science and Management, Penn State University, 202 Forest Resources Building, University Park, PA, 16802 USA; 32 Department of Plant Sciences, University of California Davis, One Shields Ave., Davis, CA 95616 USA; 33 Department of Entomology, University of Wisconsin, 1630 Linden Dr., Madison, WI 53706 USA; 34 Unit for Sustainable Forest and Environmental Management, University of Santiago de Compostela, 27002 Lugo, Spain; 35 USDA Forest Service, Southern Research Station, PO Box 1270, Hot Springs, AR 71901 USA; 36 USDA Forest Service, Rocky Mountain Research Station, 2500 S Pine Knoll Dr, Flagstaff, AZ 86001 USA; 37 Spatial Informatics Group, LLC, 2529 Yolanda Court, Pleasanton, CA 94566 USA; 38 USDA Forest Service, Southern Research Station, 320 Green Street, Athens, GA 30602-2044 USA; 39 Natural Resources Canada-Canadian Forest Service, Pacific Forestry Centre, 506 West Burnside Rd, Victoria, British Columbia V8Z 1M5 Canada; 40 USDA Forest Service, Pacific Northwest Research Station, Threats Characterization and Management Program, 1133 N. Western Ave, Wenatchee, WA 98801 USA; 41 USDA Forest Service, Sustainable Forest Management, 201 14th St SW Mailstop 1115, Washington, DC 20024 USA; 42 Department Entomology, University of Wisconsin-Madison, 1630 Linden Drive, Madison, WI 53706 USA; 43 California Department of Forestry and Fire Protection, Fire and Resource Assessment Program, 3141 US 50, South Lake Tahoe, CA 96155 USA; 44 Woods Hole Research Center, 149 Woods Hole Road, Falmouth, MA 02540 USA; 45 FireTree Wildland Fire Sciences, LLC, 523 E. Central Ave, Missoula, MT 59801 USA; 46 Department of Environmental Science and Policy, University of California, Davis, Davis, CA 95616 USA; 47 Department of Ecology, Evolution and Marine Biology, University of California, Santa Barbara, CA 93106 USA; 48 National Institute for Occupational Safety and Health, Centers for Disease Control and Prevention, 315 E. Montgomery Ave., Spokane, WA 99207 USA; 49 USDA Forest Service, Region 5, State and Private Forestry, 1323 Club Drive, Vallejo, CA 94592 USA; 50 National Park Service, Yellowstone National Park, PO Box 168, 22 Stable Street, Yellowstone National Park, WY 82190 USA; 51 New Mexico State Forestry, HC 75, Box 100, Chama, NM 87520 USA; 52 U.S. Geological Survey, New Mexico Landscapes Field Station, 301 Dinosaur Tr., Santa Fe, NM 87508 USA; 53 USDA Forest Service, Pacific Northwest Research Station, 3200 Jefferson Way, Corvallis, OR 97331 USA; 54 USDA Forest Service, Rocky Mountain Research Station, Wildland Fire Management Research Development and Application, 63095 Deschutes Market Road, Bend, OR 97701 USA; 55 Department of Geography, Portland State University, 1721 SW Broadway, Portland, OR 97201 USA; 56 The Nature Conservancy, 114 N., San Francisco Street #205, Flagstaff, Arizona 86001 USA

**Keywords:** Fire ecology, Ecological modelling

## Abstract

Wildland fires have a multitude of ecological effects in forests, woodlands, and savannas across the globe. A major focus of past research has been on tree mortality from fire, as trees provide a vast range of biological services. We assembled a database of individual-tree records from prescribed fires and wildfires in the United States. The Fire and Tree Mortality (FTM) database includes records from 164,293 individual trees with records of fire injury (crown scorch, bole char, etc.), tree diameter, and either mortality or top-kill up to ten years post-fire. Data span 142 species and 62 genera, from 409 fires occurring from 1981-2016. Additional variables such as insect attack are included when available. The FTM database can be used to evaluate individual fire-caused mortality models for pre-fire planning and post-fire decision support, to develop improved models, and to explore general patterns of individual fire-induced tree death. The database can also be used to identify knowledge gaps that could be addressed in future research.

## Abstract (summary)

**Table:** 

Measurement(s)	plant morphology trait • tree mortality • fire • tree fire injury • wildfire
Technology Type(s)	digital curation
Factor Type(s)	year of data collection • geographic location of fire • tree fire injury
Sample Characteristic - Organism	trees
Sample Characteristic - Environment	forest ecosystem
Sample Characteristic - Location	Cascades Region • Blue Mountains • Far Northern Rockies • Sierra Nevada • Piedmont Province • Region of Piedmont • Atlantic and Gulf Coastal Plain Floristic Province • Northern Rocky Mountains Provincial Park

Machine-accessible metadata file describing the reported data: 10.6084/m9.figshare.12369293

## Background & Summary

Wildfires burn millions of forested hectares annually, influencing regional and global carbon storage, wildlife habitat, hydrology, species diversity, and forest structure, along with human society and economy. Wildland fires directly kill trees, but also interact with other stressors and disturbances to cause additional delayed tree mortality^1^. The impact of a fire on a forest ecosystem (i.e., fire severity) is often quantified by the proportion of fire-caused tree mortality. Likewise, the severity of a fire regime—the aggregated impact of many fires over time—is often described by the range of variability in proportion of trees killed by fire^2,3^. Because of the economic and ecological importance of fire-caused tree mortality, a great deal of work has gone into developing predictive models of mortality and integrating those models into decision support systems for management^4,5^. The most commonly utilized models are based on empirical data: field observations of fire injury and subsequent individual tree mortality in the years following fire. Sometimes injury from fire is measured directly (e.g., crown scorch), while other measurements may be a proxy for injury that can be quickly assessed (e.g., char on bark as a proxy for cambium injury). Measurements of fire-caused injuries used in many individual tree mortality models include percentage crown volume scorched, percentage crown length scorched, percentage crown volume killed, bark char height, and cambium kill rating^5–8^. Many models also use measurements of tree resistance to fire, particularly bark thickness, which scales positively with tree diameter but at different rates among species^4,5^.

The most commonly implemented empirical model predicting post-fire tree mortality was developed by Ryan and Reinhardt^9^ and amended by Ryan and Amman^10^. This model relies on three parameters to predict probability of mortality within three years of a fire: tree species, injury to the tree crown (in the form of percentage volume of crown scorched by fire), and tree diameter (used to calculate bark thickness). This model has been implemented in many decision support systems predicting post-fire tree mortality, including the First Order Fire Effects Model (FOFEM)^11,12^, the Fire and Fuels Extension to the Forest Vegetation Simulator (FFE-FVS)^13^, and BehavePlus^14^. Within these decision support systems, the model predicts probability of tree mortality. No differentiation is made between obligate seeders and species capable of resprouting; therefore, mortality predictions are more accurately top-kill predictions for resprouting species. Additional models have been developed that account for species’ unique fire resistance traits (e.g., protected buds^15,16^), biotic consumers^7,17,18^, and abiotic stress^19–21^. Models of post-fire tree mortality and top-kill in landscape-scale models and Dynamic Global Vegetation Models (DGVMs) generally employ simplified approaches to modeling fire injury, but still rely on plant functional traits, such as bark thickness, to make mortality predictions for species’ groups^22–24^.

**Fig. 1:**
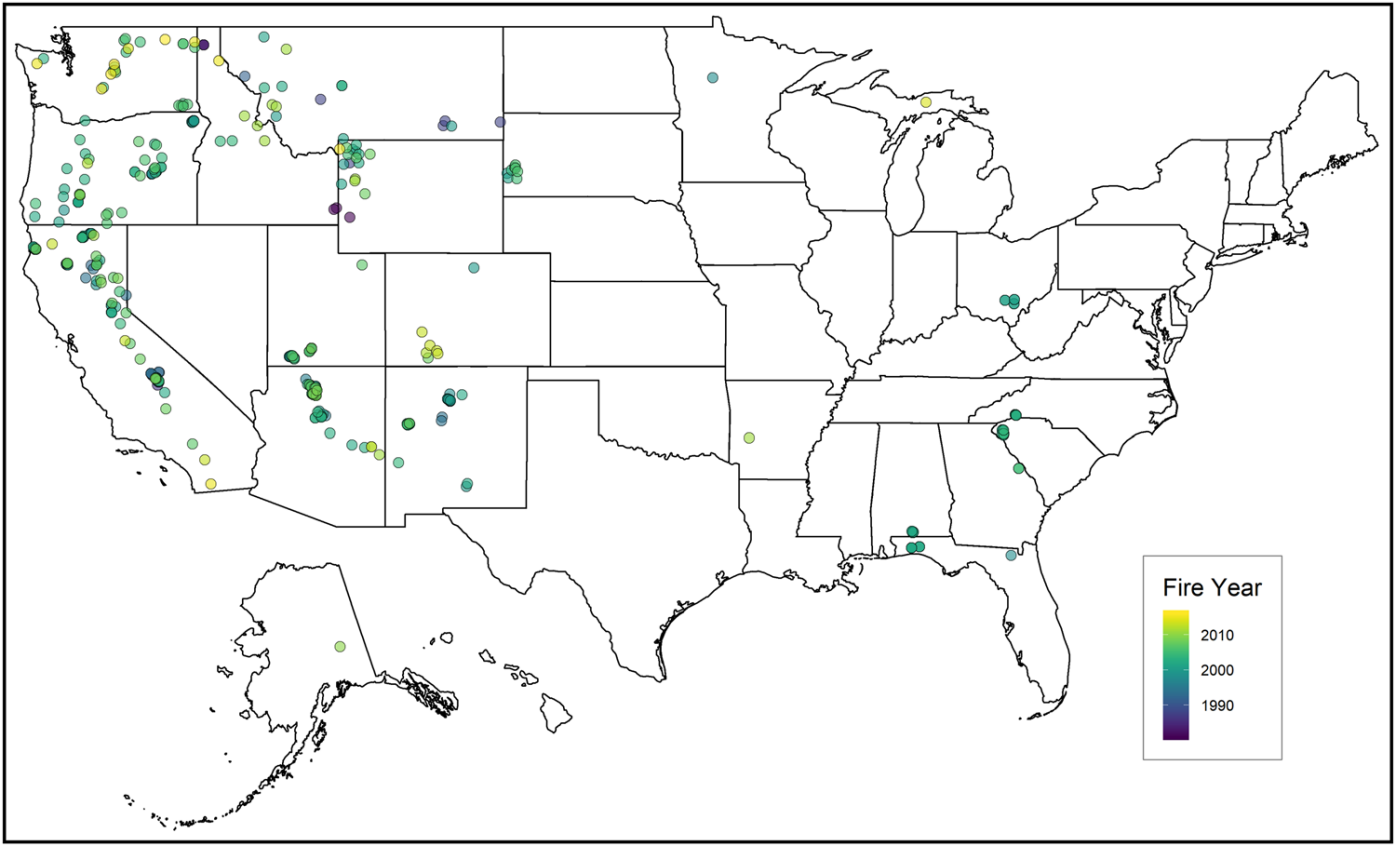
Map of fire locations by year of occurrence in the Fire and Tree Mortality (FTM) database. If a site burned twice, only the most recent fire is shown.

There have been numerous studies conducted to improve ecological understanding of the many factors that contribute to post-fire tree mortality, and to build predictive models with greater accuracy^4,5^. In an effort to capture the data from these individual studies to facilitate more expansive analyses and to identify knowledge gaps, we assembled the largest and most comprehensive collection of observations of fire-caused individual tree mortality and top-kill in the United States, the Fire and Tree Mortality (FTM) database (10.2737/RDS-2020-0001)^25^ (Fig. 1). The purpose of the FTM database is to provide access to data on individual tree mortality or top-kill from wildland and prescribed fire. The FTM database allows for large-scale evaluation of existing post-fire-mortality models over large geographic and climatic ranges for numerous species. Observational data cover the full range of fire injuries and a large proportion of tree sizes for many species, but they also reveal where data are scant or non-existent. By pooling individual datasets and ensuring comparability among variables, it becomes feasible to explore general patterns of fire-induced tree death and top-kill, to develop improved models, and to identify data gaps to inform future research.

## Methods

### Soliciting data contributions

To construct our FTM database, authors Cansler, Hood, Varner, and van Mantgem conducted a literature search for publications reporting on post-fire tree mortality and contacted corresponding authors, related investigators, and managers to inquire if they were willing to contribute data. We also posted data requests on electronic mailing lists, professional management and science exchange networks, and with technical working groups. We identified and obtained archived datasets or entered them manually from archived copies. Lastly, we coordinated with the National Park Service fire ecology program to include the agency’s Fire Effects Monitoring data^26^.

### Data aggregation and standardization

We developed the FTM database with standardized field observations from 41 contributed databases from researchers, managers, and archived datasets. Some datasets already contained aggregated data from more than one previous study^27,28^. At a minimum, datasets had to contain measurements of individual trees, stem diameter, fire injury, and post-fire status of above-ground stems (i.e., alive or dead). Post-fire injury measurements were collected either in the same season of the fire, or one to two years after fire. Tree diameter and height measurements were recorded either before the fire, or one to two years after fire. For the majority of cases, status of aboveground stems was recorded one to three years after fire; for some trees, status was re-evaluated in the years following fire. A tree or stem was considered dead when no green foliage remained in the crown. For obligate-seeding species, tree status almost always represents the true status of the individual: when the main stem dies, the tree dies. The exception is where the stem splits at or below breast height (BH, 1.37 m); in this situation, stems are considered separate trees, each with its own status. For species that resprout from the base or root structures, tree status in the FTM database represents survival of the main stem (i.e., top-kill). Resprouting from below-ground structures or above-ground epicormic buds are not captured in the database. We included any tree where post-fire status was measured within 10 years of the fire, noting the post-fire year(s) of status assessment. Only trees that were recorded as alive before the fire were included in the database. Many datasets included variables beyond the required minimum; we retained many variables on fire-caused injuries and biotic agents from the original datasets.

**Fig. 2:**
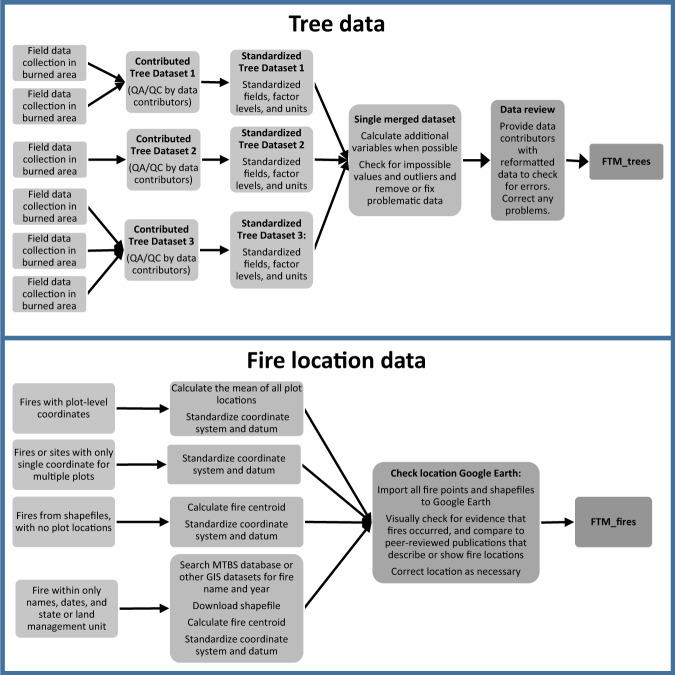
Data collection and processing workflows for individual-tree data and fire-location data used to generate the data outputs. For individual-tree data, we performed quality control measures and developed standardized fields and data from 41 contributed datasets (i.e., datasets 1 through 41). At a minimum, contributed datasets had to contain measurements of individual trees, stem diameter, fire injury, and post-fire status of above-ground stems (i.e., alive or dead). Contributed datasets sometimes contained observations from multiple fires, sites, or studies. Contributed datasets contained post-fire injury measurements and tree status collected either in the same season of the fire, or one to two years after fire. In some datasets tree status was re-evaluated in multiple years following fire. We then combined tree data into a single file. Because locations in contributed datasets were provided at different scales (e.g., tree, plot, research site, and fire) we standardized all location data in the FTM database to the scale of a fire event. QA/QC = Quality assurance and quality control. MTBS = Monitoring Trends in Burn Severity https://www.mtbs.gov.

For all contributed datasets, we verified and changed all variable names and units for consistency and labelled the levels of categorical variables. We used summary tables and data visualization to identify outliers, impossible values, and duplicate records. We corresponded with data contributors when additional clarification was needed (Fig. 2). Because many of the contributed datasets were used previously for research, error checking and quality control procedures (QA/QC) had been conducted on much of the data prior to transfer to this project. For most datasets, few errors were found during the QA/QC process. Two large datasets from the National Park Service Fire Effects Monitoring Program^26^ and the Fire and Fire Surrogate Study^28^ contained longitudinal data from many sites. In these datasets, we corrected more errors after extensive checking. In the NPS dataset, we identified and removed individual tree records that were likely duplicates. For example, where two records in the same plot shared the same tag number and species, and a similar tree diameter at breast height ("DBH"; 1.37 m above ground), one record was dropped. Likewise, in the Fire and Fire Surrogate dataset (particularly from sites in the Southern USA), some tagged trees were identified as different species in sequential measurements. In these instances we retained the most recent species code, assuming that identities were corrected over time. For all datasets, we enforced consistency in coding of status (live/dead). If a tree was alive in the final assessment year, it was coded as live in previous years. If a tree was dead, it was coded as dead in subsequent years. If a tree re-burned in a second fire and post-fire injury and status information were available following the fire, a new record (row) was made for the tree after the second fire. Database contributors were able to check and offer corrections following the data standardization procedures.

### Standardization of taxa

We standardized all scientific nomenclature and species acronyms to follow the PLANTS Database http://plants.usda.gov. Data represent 142 species and 62 genera (Online-only Table 1). Some trees were unidentified or identified only to genus (14 genera; Online-only Table 1). In some instances, trees were identified to genus, but data contributors noted that the tree could be only one of two species. In total, there are three such identifiers: *Abies grandis* or *A. lasiocarpa, Pinus jeffreyi* or *P. ponderosa*, and *Picea pungens* or *P. engelmannii* (Online-only Table 1). Finally, some contributed datasets contained unidentified trees that were alive before the fire. We retained those records and with them, a code for “unknown tree”, but we caution that unidentified trees may have been removed from other datasets during earlier quality control steps. In total, the FTM database has 161 unique tree identifier codes.

### Calculating injury variables

The FTM database includes several tree injury variables (Table 1). If variables were measured or visually estimated in the field, then we used field-based observations rather than calculated values. When these variables were not measured in the field, if possible, we calculated derived variables from those measured in the field. Specifically, we calculated:

1 $$C{L}_{pre}=HT-HC{B}_{pre}$$

where $C{L}_{pre}$ is pre-fire crown length (m), $HT$ is tree height (m), and $HC{B}_{pre}$ is the pre-fire height of the base of the crown. If $HT$ or *HCB* were measured before the fire, we used pre-fire height and crown base height. Otherwise, $HT$ and $CB{H}_{pre}$ were measured post-fire, either the season after the fire or within two years. $HT$ measurements taken more than two years after the fire were coded as “NA” (not available) and were not used. Studies have established the validity of reconstructing the pre-fire living portion of the crown after fire to estimate pre-fire height and crown base height^29,30^.

**Table 1:** FTM_trees injury-variable names and descriptions.

Variable	Description
CL_m	Pre-fire live crown length rounded to the nearest 0.01 meter.
HT_m	Either the pre-fire tree height, or if pre-fire height is not measured, the post-fire tree height taken at that same time that fire-injury variables were measured; values are rounded to the nearest 0.01 meter.
HCB_post	Post-fire height to live crown base rounded to the nearest 0.01 meter.
CR_post	Post-fire live crown ratio. Crown length divided by tree height (proportion rounded to the nearest 0.01).
CSH_m	Height of crown scorch, assessed as the highest visible heat injury to leaves from ground level, rounded to the nearest 0.01 meter. Includes scorched and consumed portions of the crown.
CLS_m	Length of the pre-fire crown that was scorched or consumed by fire rounded to the nearest 0.01 meter.
CLK_m	Length of the pre-fire crown for which fire killed tree buds by scorch or consumption, rounded to the nearest 0.01 meter.
CLS_percent	Percentage of the pre-fire crown length that was scorched or consumed by fire rounded to the nearest 1.0 percent (ranges from 0 to 100).
CLK_percent	Percentage of the pre-fire crown that was scorched, resulting in bud kill or consumption by fire, rounded to the nearest 1% (ranges from 0 to 100).
CVS_percent	Percentage of the pre-fire crown volume that was scorched or consumed by fire (ranges from 0 to 100).
CVS_percent_source	Denotes whether directly assessed in the field or derived as described in FOFEM help document. F = field; C = calculated.
CVK_percent	Percentage of pre-fire crown volume killed by fire (range of 0 to 100).
CVK_percent_source	Denotes whether directly assessed in the field or derived as described in FOFEM help document. F = field; C = calculated.
CVC_percent	Percentage crown volume consumed or blackened by the fire (range of 0 to 100).
CBS	Percentage of the circumference of the bole that was scorch (ranges from 0 to 100).
BCHA_m	Average bark char vertical height from the ground on a tree bole, rounded to the nearest 0.01 meter. Heights were visually estimated or computed as the mean of the maximum and minimum bark char height.
BCHM_m	Maximum bark char height from the ground on a tree bole, rounded to the nearest 0.01 meter.
BCH_percent	Percentage of tree height blackened or charred, based on the maximum bark char height (values 0 to 100).
BCA	Average bark char rating. A bark char rank value (numerical code) was given to each of four quadrants at the base of the tree, then values were averaged. If fewer than four quadrents were measured, this is the average of measered sections. Codes: 0 = unburned, 1 = light, 2 = moderate, and 3 = deep^32^.
CKR	Cambium kill rating. Cambium status (live or dead) was assessed in four quadrants of each tree. If fewer than four quadrants were measured, this is the average of measured sections. CKR is the number of quadrants with dead cambium at the ground line (ranges from 0 to 4)^32^.
GCA	Average ground char rating. Severity of soil heating (based on ground char) was assessed in four quadrants around each tree (1 = light, 2 = moderate, and 3 = heavy [or deep]). The four ratings were then averaged^100^. If fewer than four quadrants were measured, this is the average of measured sections.

Also included are variables used to calculate fire-injury variables. Most fire-injury variables were measured in the field the season or year after fire. Fire-injury variables that were derived from field-measured variables are described in the text. Full descriptions are documented in the metadata in Cansler *et al*.^25^.

Likewise:

2 $$C{L}_{post}=HT-HC{B}_{post}$$

where $C{L}_{post}$ is post-fire crown length (m), $HT$ is tree height (m), and $HC{B}_{post}$ is the post-fire height of the base of the crown.

Using the pre- and post-fire crown length, we could calculate the length and percentage of crown length scorched:

3 $$CL{S}_{meters}=C{L}_{pre}-C{L}_{post}$$

and

4 $$CL{S}_{percent}=100\left(\frac{CL{S}_{meters}}{C{L}_{pre}}\right)$$

where *CLS*_*meters*_ = crown length scorched measured in meters, *CLS*_*percent*_ = percentage crown length scorched, *CL*_*pre*_ = pre-fire crown length (m), and *CL*_*post*_ = post-fire crown length (m). If *CLS*_*meters*_ was measured in the field, we used that measurement of injury, instead of the change from pre-fire to post-fire crown base height for subsequent calculations.

For studies that separated crown injury as scorch, kill, or consumed, we included the amount of crown consumed in all calculations of crown scorch or crown kill.

For trees without observed crown volume scorched values, we followed the equation in the FOFEM Help manual^12^ (derived from Peterson and Ryan)^31^.

5 $$CV{S}_{percent}=100\left(\frac{CL{S}_{meters}(2C{L}_{pre}-CL{S}_{meters})}{C{L}_{pre}^{2}}\right)$$

where *CVS*_*percent*_ = percentage crown volume scorched, $CL{S}_{meters}$ = crown length scorched, and $C{L}_{pre}$ = pre-fire crown length. Because this calculation includes assumptions about tree crown architecture, it may introduce error. Thus in a separate column we coded whether the $CV{S}_{percent}$ value was based on field observation or derived from the canopy volume equation.

Likewise, for trees with observations of crown length killed ($CL{K}_{meters}$), but not percentage crown volume killed ($CV{K}_{percent}$), we calculated $CV{K}_{percent}$ using the same equation form as in Eq. 5, above:

6 $$CV{K}_{percent}=100\left(\frac{CL{K}_{meters}(2C{L}_{pre}-CL{K}_{meters})}{C{L}_{{pre}^{2}}}\right)$$

Where calculations produced an impossible value (<0 or >100) we assigned the value a code of “NA” (see “Usage Notes” below).

Damage to tree stems was measured in several ways. The most common method measured the amount (e.g., height, circumference, or percentage) of char on the tree’s bark (Table 1). Char is blackened residue of bark resulting from incomplete combustion and is a coarse indicator of the duration of bole exposure to flames and heat from the fire. Cambium kill rating (CKR) is an estimate of the amount of cambium kill and stem injury from fire^15,32^. Measurements of CKR require removing a small sample of bark at four locations at a tree’s base to determine if the underlying vascular meristematic tissue was killed by the fire. CKR is the number of quadrants (0-4) with dead cambium.

Presence or absence of beetles that are primary mortality agents on a given tree species are used in some species-specific post-fire mortality models^5,27^. These beetle species include *Dendroctonus ponderosae* (mountain pine beetle) on *Pinus* spp.; *D. valens* (red turpentine beetle), *D. ponderosae*, *D. brevicomis* (western pine beetle) or *Ips* spp. (engraver beetles) on *Pinus ponderosa;* and *D. pseudotsugae* (Douglas-fir beetle) on *Pseudotsuga menziesii*. Individual studies may have collected more detailed beetle-attack data, but for the FTM database, we simplified all attack data as presence or absence. Some studies noted presence or absence of primary bark beetles without identifying the species: thus, we combined all presence/absence information for identified and unidentified primary bark beetles into a single “beetle” variable. When studies identified beetles to species, we included species-level presence/absence information. We also included presence/absence information for a few beetle species that are not primary agents of mortality, but have been used as predictors in some models^27^, such as ambrosia beetle (subfamilies Scolytinae and Platypodinae) and *D. valens*.

### Tree identification, plot design, and study purpose

This database was developed for modeling tree mortality and top-kill at the individual-tree scale. In the *FTM_trees.csv* file we provide plot and tree number identification information to maintain consistency between the FTM database and the original contributed dataset. This ensures that each tree in the FTM database can be connected to its original record. Additionally, plot numbers and fire names can be used to track how observations are spatially grouped. We also provide study design information, including whether sampling was conducted at the individual-tree scale or if fixed-area or variable-radius plots were used. For fixed-area plots we define plot size and the minimum DBH sampled. For variable-radius plots, we provide the BA factor used. Finally, we provide standardized descriptions of the purpose(s) of the original studies.

### Fire locations

We standardized the fire location and year-of-fire data for all observations to a consistent datum and geographic coordinate system (GCS WGS84; Fig. 2). Because locations in contributed datasets were provided at different scales (e.g., tree, plot, research site, and fire) we standardized all location data in the FTM database to the scale of a fire event. If tree or plot coordinates were provided, we took the average of those coordinates to provide a centroid for the fire event. If only research site coordinates were provided, but multiple fires occurred with different start dates, we replicated those coordinates for each fire event. If a fire name and year were provided without associated geographic coordinates, we searched the Monitoring Trends in Burn Severity database https://www.mtbs.gov for the fire, downloaded the fire geospatial data, and used the coordinates of the centroid of the fire perimeter. In instances where fires were not large enough to be in the MTBS database and we lacked coordinates, we used fire perimeter data from the local land management agency to identify fire locations. All fire-location data were uploaded to Google Earth Pro^33^, and the available high-resolution pre-fire and post-fire imagery and Google Earth database of place names were used to verify the fire occurrence and location. Errors or discrepancies in fire locations and dates were corrected through correspondence with data contributors.

### Bark thickness coefficients

We provide data to calculate bark thickness for most of the species in the FTM database, following the method used in FOFEM 6.4. Specifically, bark thickness is estimated from a linear relationship with DBH and a species-specific barkthickness coefficient. FOFEM provides bark thickness coefficients for 192 tree species. If a species is absent, FOFEM users can substitute a species with similar bark thickness for modeling, or use one of the 24 bark-thickness relationships provided at the genus level. For species lacking a species-level bark thickness coefficient in FOFEM, we provide a coefficient from a morphologically similar species or the genus (if available). Of 159 taxa identifiers in the database, we include bark thickness coefficients for 148.

## Data Records

The FTM database is available for download from the USDA Forest Service Research Data Archive^25^. The FTM database includes standardized field observations of fire injury and survival from 164,293 individual trees. Of these, 6,670 trees have records relating to two separate fires, resulting in a total of 170,963 observations. The data span 21 states and include 409 prescribed fires and wildfires from 1981 to 2016 (Fig. 1). The data represent 142 species and 62 genera; 97.3% of the trees are identified to species and 99.7%, to genus.The archived data product consists of a metafile in both HTML and XML formats, a TIFF file showing the geographic locations of fires, and five separate data files:

- *Dataset_citations.csv*: Comma-delimited ASCII text file containing the main citation for each contributed dataset in the Fire and Tree Mortality (FTM) database.
- *Dataset_primary_contacts.csv*: Comma-delimited ASCII text file containing dataset names as they appear in the FTM database and the associated primary contact information.
- *FTM_fires.csv*: Comma-delimited ASCII text file containing fire names, year, dataset contact, and location for fires in the FTM database.
- *FTM_trees.csv*: Comma-delimited ASCII text file containing tree-level records of fire injury, tree size, and bark beetle attack.
- *Species_BarkThickness.csv*: Comma-delimited ASCII text file containing the list of species found in the FTM database and the bark thickness information used to evaluate FOFEM version 6.4 model accuracy.

**Fig. 3:**
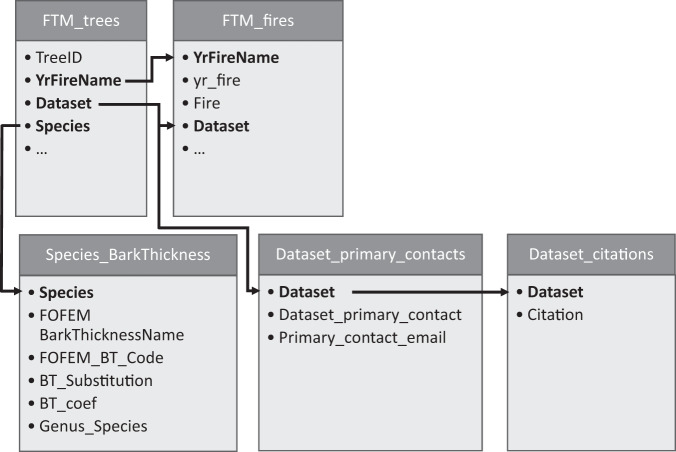
Fire and Tree Mortality (FTM) database structure showing individual files contained in the FTM database. Bold arrows indicate common fields that can be used to join files.

Figure 3 shows the common fields and connections among each of the five data files.

## Technical Validation

The data used to build the FTM database primarily come from high-quality data sources that have been used in other analyses and peer-reviewed publications, or from long-term institutional monitoring studies. The majority of individual-tree observations are derived from peer-reviewed studies^6–8,15,17,18,27,34–74^. Data were contributed by corresponding authors, or came from archived datasets from completed projects^28,40,75–78^. Twelve additional datasets were not peer reviewed, but were summarized in professional reports or theses^79–82^ or represent ongoing research or monitoring by land management professionals^26,83–89^. The contributed database with the largest sample of fires is from the National Park Service Fire Effects Monitoring Program, a long-term institutional monitoring program in which permanent field plots are resampled on a standardized schedule with trained staff and established quality controls^26^. These studies and monitoring projects were designed for a range of purposes, listed in the *FTM_Fires.csv* file, including modelling post-fire tree mortality^6,7,15,17,18,40,42,47,48,50,58,71,74,79,90–92^, understanding the effectiveness of prescribed fire at reducing fuel loading, future fire severity, restoring historical forest structure^38,39,56,57,59–61,66,67,69,82,93^, tracking post-fire successional dynamics^43,45,59,62,63,90,94^, developing remote sensing indices to understand landscape fire effects^46,70,95^, carbon emission modeling^65^, plant physiological research^36,53–55^, and research on interactions between fire and bark beetles^4,7,10,18,34,35,37,41,43,44,49,51,81^. The file *Dataset_citations.csv* provides the primary citations for each contributed dataset in the FTM database.

## Usage Notes

We developed the FTM database to validate existing models of individual-stem and tree-scale post-fire tree mortality^96^ and to support development of new models. Researchers may find additional uses for these data, but we urge caution in their use. For any use, researchers should consider possible sources of error. Despite multiple procedures for quality control, there are likely to be errors of observation and calculation present in the final FTM database. For example, many post-fire injury measurements, such as crown volume scorch, are subjective field estimates, and may vary among observers (although consistency within a study is likely to be higher than consistency across studies). Data from studies that included repeated measurements over time will be more accurate than those based on a single post-fire measurement. Common errors that can be identified and corrected through repeated measurements include misidentified species, duplicate or missing records, incorrect diameter measurements, and incorrect tree status (e.g., mistakenly identifying trees as dead). We excluded trees that were dead prior to fires, and we excluded ingrowth that reached minimum measurement sizes after the fire that were recorded in longitudinal datasets. For studies where plots were measured post-, but not pre-fire, there may be errors in pre-fire status if trees that died shortly before the fire were erroneously coded as alive. Any calculations of carbon stores from this dataset could only include pre-fire live carbon, since contributed datasets did not consistently include measurements of trees that were dead before the fire, and therefore we did not include any trees that were dead before the fire in the database.

Crown injury variables derived from field observations are also susceptible to errors. Most derived variables are based on simple calculations (detailed above) and after each calculation we checked for impossible values. If found, these were coded as “NA”. The most common error of this sort occurred when pre-fire crown base height was slightly higher than post-fire crown base height (resulting in a negative value for crown length scorch). This error likely reflects varying precision in the measurement of crown base height before and after the fire, but it could also reflect a data collection or data entry error. For crown volume scorch and crown volume killed (Eqs. 5 and 6), the equation, based on assumptions of tree crown shape and crown length, may introduce error. For transparency, we coded which observations were based on field observations and which were derived from the crown volume equation. Percentages of crown length and crown volume are positively correlated but are not the same or interchangeable^15^, and models using field-based measurements perform better^96^. In addition, users should be aware that observations of crown scorch typically imply that the scorched portions of the crown are killed by the fire (i.e., bud kill or crown kill). However, this is not always true for species with large buds or epicormic sprouting^4^. Thus, the FTM database crown scorch values should be understood to represent the proportion of the tree’s leaves that were killed by fire, but not the extent of bud mortality or the potential for branch recovery. For studies that differentiated between crown scorch and crown kill levels, the percentage of crown scorched must always be greater than or equal to the percentage of crown killed.

**Fig. 4:**
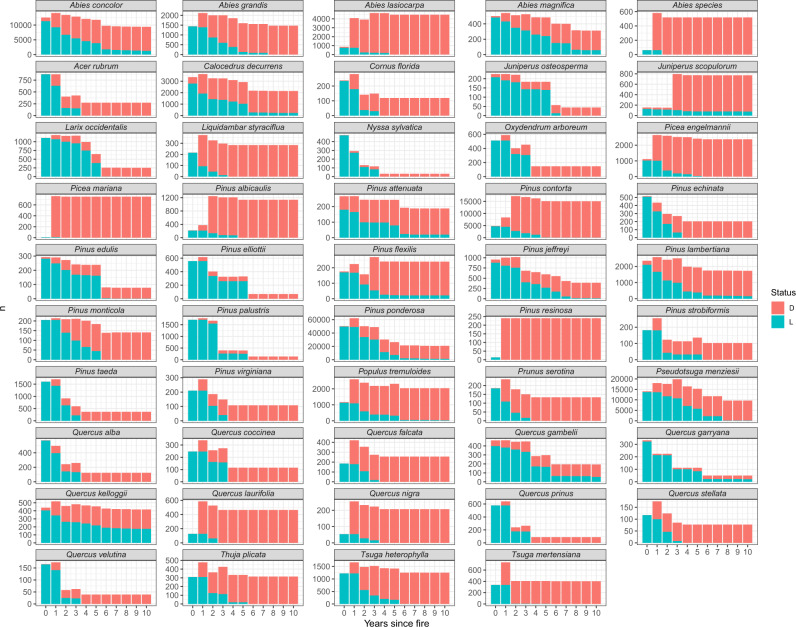
Number of tree-status observations (*n*) by years since fire for live (L) and dead (D) trees. Only species with ≥200 samples are shown. We filled in missing values for tree status when possible (e.g., dead trees remained dead after monitoring ceased; live trees were coded as live in previous years). The longer the time since fire, the more likely a database will contain only dead trees for a given species.

**Fig. 5:**
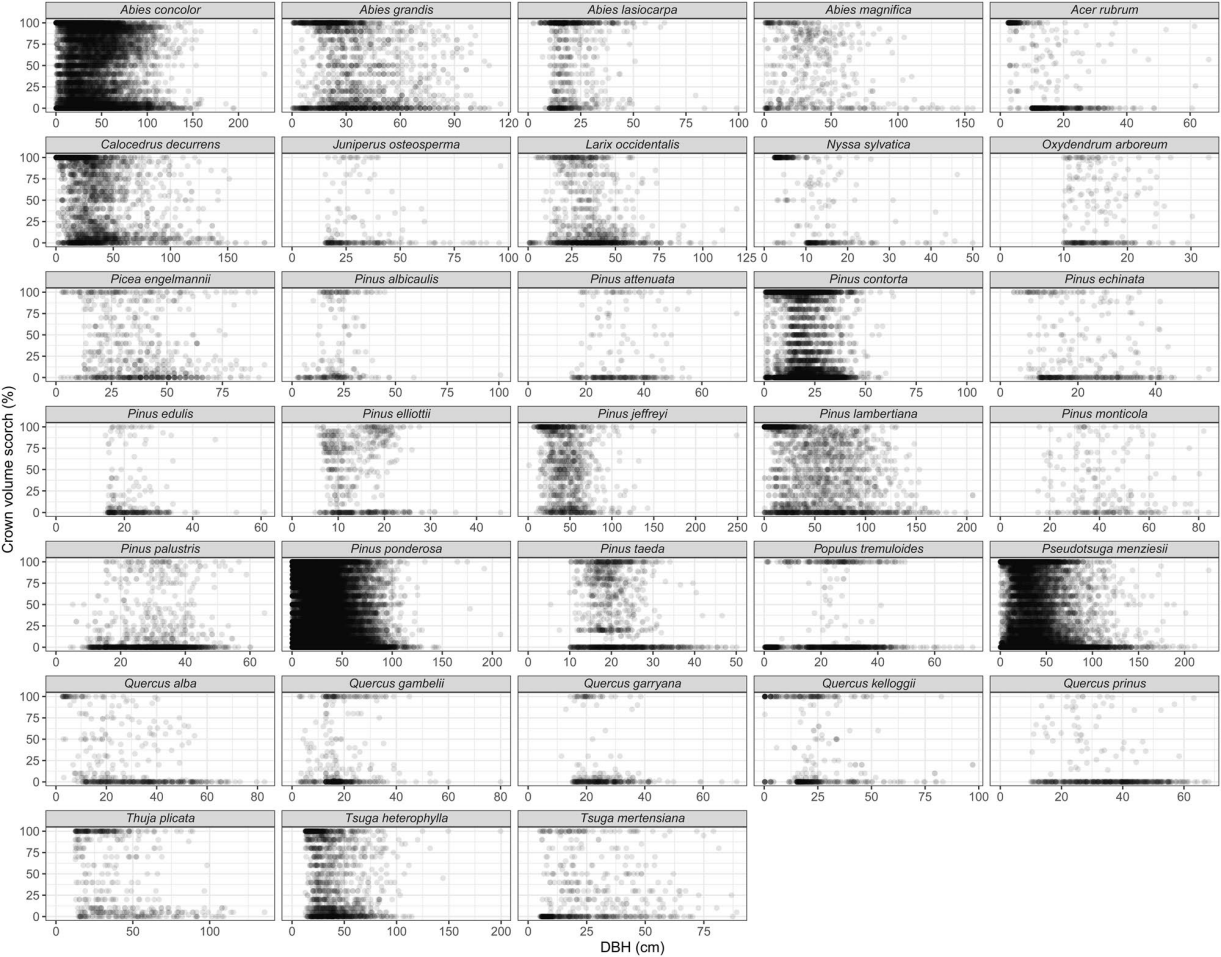
Scatterplots of tree diameter at breast height (DBH) vs. percentage crown volume scorch (CVS) for species with ≥200 observations of both variables. These data displays can show gaps in information, such as small or large trees or species for which there are few or no combinations of DBH and CVS.

For tree mortality and top-kill modelling, we note three limitations in particular. First, because different combinations of injury variables were measured in each study, there are many missing values in the FTM database. Second, tree status observations decline—particularly observations of live trees—as time since fire increases (Fig. 4). Because we extrapolated tree status for years when plots were not measured, modeling of plot-scale proportional mortality would not be an appropriate use of the data. Third, in building empirical models, it is important to consider the data range for the variables used, and not simply for the individual variables, but for the combined predictor space represented in the dataset^97^ (Fig. 5).

The FTM database includes information on sampling design, and where applicable, plot size and minimum tree diameter sampled. The best use for the fire-scale and plot-scale identifiers is understanding and accounting for the spatial aggregation or nesting of many of the observations (e.g., by using hierarchical or mixed-effects models)^98^. Although plot-level metrics, such as stem density, basal area, or stand density index can be generated from some datasets, we did not develop the FTM database explicitly for plot-level modeling. Plot-level measurements can be used to quantify variation in forest structure or intensity of competition, but with caution due to variation in how trees were sampled among studies. There are several possible types of unaccounted variation in plot-level statistics: (1) individual tree records that were incomplete and removed from contributed datasets before transfer to the current study; (2) undocumented procedures for subsampling different tree diameter classes; and (3) undocumented exclusion of species (e.g., angiosperms) or growth forms (e.g., tall woody shrubs or hardwoods) that would have influenced stem densities or indices of competition.

Plots were not the sampling units in all contributed datasets. For many studies—particularly physiology and bark beetle studies—the individual tree was the sampling unit. In other studies, plots were used to structure the sampling, but not all trees were measured within a plot (e.g., only the first three stems of a given species or size class were sampled or only a particular species was sampled). When trees were fully censused within a plot, the minimum DBH differed among studies (noted in the *FTM_fires.csv* file), thus cross-study comparisons of plot-level statistics must be made with caution.

Because the FTM database was developed to support individual-tree scale modeling, we devoted considerable effort to identifying incomplete or duplicate records. These were detectable only in datasets with repeated measurements (e.g., National Park Service datasets). However, optimizing for complete and non-duplicate records may produce erroneous plot-level metrics (e.g., tree density). Finally, for all datasets, tree locations within plots were not recorded, thus indices of neighborhood competition at the individual-tree scale (or any finer scale than the plot) cannot be calculated. Users can refer to the primary literature contained in *Dataset_citations.csv* for additional information on study designs and dataset contents.

Pooling data from across the United States incorporates taxa that are not well represented in previous studies, such as junipers and oaks. Nevertheless, geographic and taxonomical gaps remain. Data are primarily from the western USA, with some representation of the southeastern USA (Fig. 1). Gymnosperms are better represented than angiosperms (Online-only Table 1). We encourage researchers to identify geographic or taxonomic gaps in the existing data and to target sampling to fill those gaps. We plan to update the FTM database as additional data are collected and made available from the USA and internationally.

## Data Availability

Fire and Tree Mortality Database (FTM) is available from Forest Service Research Data Archive 10.2737/RDS-2020-0001. All reformatting of contributed data was completed in R version 3.6.1^99^. Original contributed data are only available by contacting data contributors. The code used to reformat the data may be obtained contacting C. A. Cansler.

## Online-only Table

**Online-only Table 1:** Species represented in the FTM database, including number of records (with separate records for trees burned in two fires), individual trees, fire observations, and unique years in which fires occurred. In some instances, trees were identified to genus, but data contributors noted that the tree could be only one of two species.

*Species*	Records	Trees	Fires	Years
*Abies amabilis*	111	111	1	1
*Abies concolor*	14,389	14,175	109	28
*Abies grandis*	2,168	2,167	19	11
*Abies grandis* or *A. lasiocarpa*	453	453	1	1
*Abies lasiocarpa*	5,070	4,441	40	14
*Abies magnifica*	552	552	13	10
*Abies* species	581	532	1	1
*Acer floridanum*	1	1	1	1
*Acer grandidentatum*	4	4	1	1
*Acer macrophyllum*	7	7	4	2
*Acer rubrum*	1,275	1,275	23	5
*Acer saccharum*	185	185	5	1
*Aesculus glabra*	2	2	2	1
*Ailanthus* species	1	1	1	1
*Albizia julibrissin*	1	1	1	1
*Alnus incana*	60	60	2	2
*Alnus rubra*	5	5	1	1
*Amelanchier arborea*	34	34	2	2
*Aralia spinosa*	9	9	1	1
*Arbutus menziesii*	19	19	4	2
*Asimina triloba*	8	8	1	1
*Betula lenta*	3	3	3	1
*Calocedrus decurrens*	3,654	3,483	43	17
*Carpinus caroliniana*	13	13	4	2
*Carya glabra*	20	20	9	5
*Carya pallida*	17	17	5	2
*Carya* species	127	127	8	2
*Carya texana*	89	89	1	1
*Carya tomentosa*	151	151	16	5
*Castanea dentata*	1	1	1	1
*Castanea pumila*	2	2	1	1
*Celtis laevigata*	2	2	1	1
*Celtis occidentalis*	7	7	1	1
*Cercis canadensis*	10	10	3	2
*Chamaecyparis lawsoniana*	69	69	2	2
*Chamaecyparis nootkatensis*	26	26	1	1
*Chrysolepis chrysophylla*	7	7	2	1
*Cornus florida*	357	357	27	5
*Cornus nuttallii*	119	119	5	3
*Crataegus marshallii*	1	1	1	1
*Crataegus* species	4	4	2	2
*Diospyros virginiana*	94	94	10	3
*Fagus grandifolia*	22	22	7	2
*Frangula caroliniana*	5	5	1	1
*Fraxinus americana*	37	37	1	1
*Fraxinus pennsylvanica*	1	1	1	1
*Fraxinus* species	14	14	4	1
*Gleditsia triacanthos*	3	3	1	1
*Hamamelis virginiana*	15	15	1	1
*Holodiscus discolor*	3	3	1	1
*Ilex opaca*	40	40	8	3
*Juglans nigra*	5	5	5	2
*Juniperus deppeana*	142	141	10	6
*Juniperus monosperma*	21	21	7	5
*Juniperus occidentalis*	72	72	5	4
*Juniperus osteosperma*	225	225	20	12
*Juniperus scopulorum*	807	499	21	11
*Juniperus virginiana*	61	61	9	4
*Larix occidentalis*	1,189	1,189	19	12
*Liquidambar styraciflua*	533	533	10	4
*Liriodendron* species	92	92	6	1
*Liriodendron tulipifera*	151	151	15	4
*Magnolia fraseri*	14	14	4	1
*Magnolia grandiflora*	37	37	2	1
*Magnolia tripetala*	10	10	1	1
*Magnolia virginiana*	8	8	1	1
*Morus rubra*	2	2	1	1
*Morus* species	1	1	1	1
*Notholithocarpus densiflorus*	127	127	4	2
*Nyssa sylvatica*	516	516	26	5
*Ostrya virginiana*	131	131	1	1
*Oxydendrum arboreum*	628	628	20	4
*Persea borbonia*	2	2	1	1
*Picea engelmannii*	2,996	2,876	45	18
*Picea glauca*	46	46	1	1
*Picea mariana*	760	760	1	1
*Picea pungens*	142	142	2	1
*Picea pungens* or *P. engelmannii*	9	9	1	1
*Picea sitchensis*	22	21	1	1
*Pinus albicaulis*	1,297	834	15	8
*Pinus arizonica*	1	1	1	1
*Pinus attenuata*	268	268	7	5
*Pinus banksiana*	1	1	1	1
*Pinus contorta*	18,298	11,615	46	18
*Pinus coulteri*	182	182	1	1
*Pinus echinata*	748	748	16	5
*Pinus edulis*	292	292	28	15
*Pinus elliottii*	634	634	5	3
*Pinus flexilis*	373	353	11	7
*Pinus glabra*	17	17	5	1
*Pinus jeffreyi*	1,068	1,068	26	14
*Pinus jeffreyi* or *P. ponderosa*	2,720	2,720	7	5
*Pinus lambertiana*	2,592	2,549	54	21
*Pinus monophylla*	3	3	1	1
*Pinus monticola*	214	214	11	8
*Pinus palustris*	1,957	1,957	18	3
*Pinus ponderosa*	64,825	61,097	260	27
*Pinus resinosa*	254	254	2	2
*Pinus rigida*	35	35	6	1
*Pinus sabiniana*	3	3	1	1
*Pinus* species	35	35	11	5
*Pinus strobiformis*	283	283	5	5
*Pinus strobus*	44	44	9	4
*Pinus taeda*	2,236	2,236	16	4
*Pinus virginiana*	370	370	13	4
*Platanus occidentalis*	3	3	1	1
*Platanus* species	4	4	1	1
*Populus deltoides*	185	185	2	1
*Populus grandidentata*	10	10	4	1
*Populus tremuloides*	2,648	2,647	41	19
*Prunus americana*	21	21	1	1
*Prunus emarginata*	2	2	1	1
*Prunus mexicana*	2	2	1	1
*Prunus serotina*	334	334	15	4
*Prunus* species	4	4	1	1
*Prunus umbellata*	6	6	1	1
*Pseudotsuga macrocarpa*	1	1	1	1
*Pseudotsuga menziesii*	21,027	20,157	111	25
*Quercus alba*	710	710	23	5
*Quercus chrysolepis*	45	45	10	8
*Quercus coccinea*	371	371	20	4
*Quercus falcata*	448	448	14	4
*Quercus gambelii*	496	493	24	13
*Quercus garryana*	333	333	5	4
*Quercus incana*	7	7	3	1
*Quercus kelloggii*	550	538	29	13
*Quercus laevis*	61	61	3	1
*Quercus laurifolia*	638	638	6	1
*Quercus marilandica*	11	11	3	2
*Quercus muehlenbergii*	1	1	1	1
*Quercus nigra*	266	266	12	3
*Quercus phellos*	1	1	1	1
*Quercus prinus*	731	731	15	3
*Quercus rubra*	162	162	16	5
*Quercus* species	59	59	8	3
*Quercus stellata*	237	237	14	5
*Quercus velutina*	230	230	19	5
*Quercus wislizeni*	7	7	2	2
*Rhus copallinum*	8	8	1	1
*Robinia pseudoacacia*	17	17	7	3
*Salix scouleriana*	3	3	2	2
*Salix* species	10	9	3	3
*Sassafras albidum*	174	174	8	3
*Sassafras* species	65	65	5	1
*Sequoia sempervirens*	7	7	1	1
*Sequoiadendron giganteum*	134	134	16	10
*Taxus brevifolia*	6	6	2	1
*Thuja plicata*	477	477	5	3
*Tilia americana*	25	25	4	3
*Torreya californica*	2	2	1	1
*Tsuga canadensis*	21	21	4	1
*Tsuga heterophylla*	1,674	1,673	14	7
*Tsuga mertensiana*	735	687	2	2
*Ulmus alata*	108	108	4	3
*Ulmus americana*	9	9	2	2
*Ulmus rubra*	2	2	2	2
*Unknown* species	500	405	21	9
*Vaccinium arboreum*	25	25	1	1
*Viburnum rufidulum*	3	3	1	1
